# The role of vaccination and healthcare acquisition in respiratory failure during a COVID-19 outbreak in an acute hospital in Wales

**DOI:** 10.1016/j.infpip.2025.100474

**Published:** 2025-07-09

**Authors:** Craig Hogg, Caoimhe McKerr, Noel Craine, Dafydd Williams, Malorie Perry, Simon Cottrell

**Affiliations:** a Communicable Disease Surveillance Centre, Public Health Wales, Cardiff, Wales, UK; b Infection Prevention Department, Ysbyty Gwynedd, Betsi Cadwaladr University Health Board, Bangor, Wales, UK

## Abstract

This study provides descriptive analysis of a COVID-19 outbreak of 323 cases in a Welsh hospital in early 2021, with a focus on respiratory failure (RF) as a severe outcome. Multivariate analysis demonstrated decreased odds of RF in healthcare acquired cases (aOR 0.40 (95%CI 0.24–0.65), *p*<0.001) and individuals with at least one dose of vaccine 14 days before first positive test (aOR 0.45 (95%CI 0.28–0.75), *p*=0.002). In healthcare acquired cases, vaccination significantly reduced the odds of RF (OR 0.47 (95%CI 0.22-0.00), *p*=0.037). This analysis highlights the importance of vaccination as a protective factor against severe respiratory outcomes.

## Background

Between April 2020 and April 2022, over 800,000 COVID-19 patients were admitted to hospitals in the UK [1,2]. Medical settings are a source of outbreaks, driven by a mix of community acquired illness and healthcare acquired infections (HCAI). Advancing age and chronic disease are associated with increased risk of severe disease, with hospital inpatients particularly vulnerable to poor outcomes [3,4].

The COVID-19 vaccination programme in Wales began on 8th December 2020, prioritising those aged 70 years and over, frontline health and social care staff, care home staff and residents and those who are extremely vulnerable. Nearly 90% of people in these groups were vaccinated by 12 February 2021, with the aim to vaccinate the rest of the adult population by autumn 2021 [1].

Between 14 January 2021 and 22 April 2021, 323 patients at a large, acute hospital site and three peripheral community hospitals in Wales were reported as part of a health board-wide COVID-19 outbreak. This outbreak occurred at a time when the universal COVID-19 vaccination programme was being rapidly rolled out to the Wales population (initially, a two-dose course), beginning with those who were most vulnerable in terms of age and clinical risk.

## Aims

The aim of this study was to characterise the incidence and determinants of respiratory failure among hospitalised patients during this outbreak, with focus on the effect of vaccination and healthcare acquisition. The analysis aims to understand the impact of these determinants on respiratory failure as a severe respiratory outcome in the context of an acute outbreak in the hospital setting, improving understanding of respiratory disease progression in outbreak cases.

## Methods

### Case definitions

A case was a patient admitted to the hospital of interest or associated community hospitals, who had a SARS-CoV-2 PCR positive sample collected on a hospital site between 14^th^ January 2021–22^nd^ April 2021. A healthcare acquired infection (HCAI) was confirmed as a first positive sample more than seven days following admission, in line with ECDC definitions [4]. Cases with a sample taken seven days or fewer following admission were categorised as community cases.

### Outcome definitions

Respiratory failure as an outcome was defined as the requirement of oxygenation due to respiratory failure within 14 days of admission. Where no severity data were available (14/323 cases [4.3%]), cases were included in descriptive statistics but excluded from further analysis.

### Case and exposure data

Cases were identified from the Laboratory Information Management System (LIMS) which records results for each laboratory test undertaken in Wales. A linelist was extracted by Public Health Wales personnel and supplemented with relevant clinical and demographic data using the local case management system. Infection prevention and control teams retrospectively reviewed patients who tested positive for SARS-CoV-2 and collected data on outcomes including oxygen requirements and respiratory failure and retained in a spreadsheet. Vaccination status, dosage, and dates were extracted from the Welsh Immunisation System (WIS), which is a registry of COVID-19 vaccination data for the entire Wales population, provided by Digital Health and Care Wales (DCHW). Cases were recorded as vaccinated if they had received at least one dose of vaccine at least 14 days prior to their first SARS-CoV-2 positive sample. Where no vaccination record was available, cases were considered unvaccinated. Length of stay was calculated using either the natural end of hospital admission or date of death where cases died during their admission to hospital.

### Deduplication and missing records

Where a patient was tested twice due to multiple admissions or repeat testing, the earliest record was retained provided the tests were within 42 days of the first positive test.

### Data handling and access

Datasets were linked through unique patient identifiers using R and stored on a secure system within the Public Health Wales usual infrastructure. Data were accessed by PHW analytical staff only, adhering to NHS Wales information governance policy.

### Data analysis

Demographic and clinical characteristics of patients were described by outcome. Age was summarised using median and interquartile range following assessment of normal distribution using histogram and Q-Q plot, and analysed as a continuous variable with odds ratios reflecting the effect of each incremental year of age. Length of stay (LOS) was dichotomised by LOS over 7 days. All other variables were treated as binary. Patient characteristics by hospital site were not detailed to avoid potential identification of individuals, given small numbers at peripheral community sites. Univariate logistic regression was conducted for each independent variable to identify possible associations with the outcome of interest. Crude odds ratios with 95% confidence intervals and *p* values were calculated for each variable to identify possible associations. Variables identified in univariate analysis with *p* < 0.2 were taken forward to multivariate analysis. Additionally, age was included due to strong statistical associations with key characteristics in descriptive analysis. Adjusted odds ratios (OR)s with 95% confidence intervals (CIs) were calculated to quantify the association between exposures and the outcome of interest in both univariate and multivariate models. Mantel-Haenszel adjusted odds ratios were calculated and presented with 95% confidence intervals and *p* values to quantify the stratified effect of vaccination status by healthcare acquisition. Analyses were undertaken using R version 4.3.2.

### Ethics statement

Public Health Wales was established under The Public Health Wales National Health Service Trust (Establishment) Order 2009. Its functions include the provision and management of a range of public health, health protection, healthcare improvement, health advisory, child protection and microbiological laboratory services and services relating to the surveillance, prevention and control of communicable diseases. This work falls under PHW core activities of outbreak response and surveillance, and no further NHS research permissions were required. Data were held and processed under Public Health Wales information governance arrangements and Public Health Wales guidance on the release of small numbers. All methods were performed in accordance with the relevant guidelines and regulations. No data identifying protected characteristics of an individual were released outside Public Health Wales.

## Findings

The outbreak took place over a 14-week period beginning in January 2021. A total of 323 cases were identified. Cases were identified in one acute hospital and on three additional community sites, spanning a total of 29 wards. The majority (96.9%) of cases were identified in the acute hospital site. Overall, 151 cases (46.7%) were determined to be healthcare acquired infections (HCAI).

Overall, 51% (n=165) of this cohort were female. The median age of cases was 77 years (range 10–99 years). Female cases (median: 79) were of a similar age to male cases (median: 77) (Mann Whitney U test; *p* = 0.86). Community acquired cases tended to be younger, with a median age of 75, compared to 81 in HCAI cases (Mann Whitney U test; *p* < 0.001).

Of 323 cases, 150 (46.6%) were vaccinated with one, or two doses at least 14 days prior to their first positive sample. Seven (4.7%) had received two doses at least 14 days prior to their first positive sample. The remaining 173 (53.5%) had no recorded vaccination history. Older cases were more likely to have been offered vaccination due to vaccination priority and age per unit was significantly associated with vaccination at 14 days prior to first positive test (Wilcoxon rank-sum, *p*<0.001). Age as a continuous variable was not significantly associated with respiratory failure (Wilcoxon rank sum; *p* = 0.155) (Table I).

**Table I:** Baseline demographic characteristics of cases, by respiratory failure (RF) outcome

Characteristic	Overall	No respiratory failure	Respiratory failure	*p*-value
	(N=309)	(N=156)	(N=153)	
**Age**				
Median [IQR]	78.0 [18.0]	80.0 [17.0]	77.0 [18.0]	0.155
**Sex**				
Female	160 (51.8%)	86 (55.1%)	74 (48.4%)	0.282
Male	149 (48.2%)	70 (44.9%)	79 (51.6%)	
**Vaccinated ≥14 days before**				
Yes	141 (45.6%)	87 (55.8%)	54 (35.3%)	<0.001
No	168 (54.4%)	69 (44.2%)	99 (64.7%)	
**Acquisition**				
Healthcare	146 (47.2%)	92 (59.0%)	54 (35.3%)	<0.001
Community	163 (52.8%)	64 (41.0%)	99 (64.7%)	
**Length of stay**				
Median [IQR]	23.0 [31.0]	26.0 [33.5]	20.0 [26.0]	0.0807
0–7 days	61 (19.7%)	30 (19.2%)	31 (20.3%)	0.933
>7 days	248 (80.3%	126 (80.8%)	122 (79.7%)	

∗*p*-values of Mann-Whitney U Test for continuous variables and Chi-squared test for categorical variables.

No significant relationship was detected between older age or sex, or length of stay in hospital and the odds of respiratory failure. (Table II).

**Table II:** Univariable and multivariate logistic regression analysis results for outcome of respiratory failure (RF) among cases, by clinical and demographic characteristics

	Unadjusted			Adjusted		
	OR	95% CI	p	OR	95% CI	p
Age	1	0.99-1.01	0.945	1.01	1.00–1.03	0.062
Sex (M)	1.31	0.84-2.05	0.235			
HCAI (Y)	0.38	0.24-0.60	<0.001	0.40	0.24-0.65	<0.001
Vaccinated (Y)	0.43	0.27-0.68	<0.001	0.45	0.28-0.75	0.002
LOS > 7 Days	0.94	0.53-1.64	0.820			

Having at least one dose of vaccine as at date of first positive sample was associated with decreased odds of respiratory failure within 14 days (OR 0.43 (95% CI 0.27–0.68), *p* < 0.001). After adjustment for age and acquisition, this effect remained statistically significant (aOR 0.45 (95%CI 0.28–0.75), *p* = 0.002).

Having a healthcare acquired infection as opposed to a community acquired infection was also associated with decreased odds of respiratory failure within 14 days (OR 0.38 (95%CI 0.24–0.60), *p* < 0.001). After adjustment for age and vaccination status, this effect remained statistically significant (aOR 0.40 (95%CI 0.24–0.65), *p* < 0.001).

Vaccination at least 14 days prior to first positive test was taken forward to stratified analysis as the primary factor of interest. Analysis was stratified by HCAI status as the only other significantly associated factor.

In stratified analysis, vaccination did reduce the odds of respiratory failure in community acquired cases, but the association was not significant at the 95% level in this study (OR 0.58 (95%CI 0.28–1.18), *p*=0.126). However, in HCAI cases, vaccination significantly reduced the odds of respiratory failure (OR 0.47 (95%CI 0.22-0.00), *p*=0.037). The Mantel-Haenszel weighted odds ratio (OR 0.52 (95%CI 0.33–0.84), *p*=0.008) demonstrates a strong overall protective effect across both strata, reinforcing the protective effect of vaccination seen in logistic regression analysis.

Those with HCAI were more likely to be vaccinated 14 days prior to first positive sample, with 59.6% of HCAI cases considered vaccinated compared with 33.1% of non-HCAI cases (Chi-squared test, *p*<0.001).

## Discussion

These findings highlight the protective effect of vaccination in an early COVID-19 hospital outbreak. This is supported by existing literature, with a significant increase in vaccine effectiveness (VE) for hospitalisation at the 14 day point [5,6]. Our findings support the use of vaccination to reduce acuity in high risk groups and to reduce risk of respiratory failure. Hospital planners can also assess vaccination uptake to anticipate respiratory support needs during periods of high prevalence.

Following this study, routine oxygenation surveillance was implemented in the health board, including COVID-19, RSV, and influenza case, allowing for monitoring of incidence and acuity. Further work could integrate vaccination and hospital episode statistics to control for comorbidities however a full VE study would require additional data linkage to mitigate confounding.

Patients with HCAIs had lower odds of respiratory failure in this study, despite being older. However, stratified analysis suggests this may be explained by higher vaccination rates in line with the vaccination strategy. Although age was included in multivariable analysis, adjusted results suggest that a larger sample size and additional confounding control is needed to fully understand this effect. Since older and comorbid patients were prioritised in the vaccination campaign, longer hospital stays may have facilitated increased opportunities for vaccination. Hospital staff and patients wearing masks may also have limited the infective dose in hospitalised patients, which some studies suggest is associated with reduced clinical severity [7,8].

The opportunistic nature of this study introduced limitations, including quality of comorbidity and wider acuity data to better control for confounding. Community acquired patients may have presented with more severe illness, whilst HCAI cases may have been incidentally identified through routine testing. Given the robust nature of Wales' vaccination records, it is unlikely that patients classes as unvaccinated may have received undocumented vaccinations, however future prospective work could incorporate overseas vaccination records to supplement the national vaccination registry.

The 7 day dichotomisation for length of stay used in regression analysis was in line with HCAI definitions and helped distinguish between short and prolonged hospitalisations. This is supported by evidence linking extended hospitalisation with poorer outcomes [9,10]. However, artificial truncation of this measure may have underestimated LOS in survivors. Further analysis of survival status may mitigate this bias in future studies.

As this outbreak occurred early in the vaccination campaign, few patients had received more than one dose. Whilst current variant circulation and booster doses limit generalisability, these findings reinforce public health messaging that partial vaccination still provides protection. The study also provides insight into clinical outcomes early in the pandemic, in conditions which are now impossible to reproduce.

This study characterises the demographic profile of patients involved in this COVID-19 outbreak and highlights the protective effect of early vaccination efforts in reducing acuity in hospital outbreaks. These findings can support clinicians and public health professionals to anticipate patient oxygenation requirements and manage outbreaks more effectively.

## CRediT authorship contribution statement

**Craig Hogg:** Conceptualization, Methodology, Software, Validation, Formal analysis, Investigation, Data curation, Writing – original draft, Writing – review & editing, Visualization, Project administration. **Caoimhe McKerr:** Conceptualization, Methodology, Software, Investigation, Data curation, Writing – original draft, Writing – review & editing, Supervision. **Noel Craine:** Conceptualization, Methodology, Writing – review & editing, Supervision. **Dafydd Williams:** Conceptualization, Investigation, Data curation. **Malorie Perry:** Methodology, Writing – review & editing, Supervision. **Simon Cottrell:** Methodology, Writing – review & editing, Supervision.

## Funding statement

The authors received no specific funding for this work.

## Conflicts of interest

The authors declare that no competing interests exist.
